# Respiratory Viral Coinfections: Insights into Epidemiology, Immune Response, Pathology, and Clinical Outcomes

**DOI:** 10.3390/pathogens13040316

**Published:** 2024-04-12

**Authors:** Pius I. Babawale, Antonieta Guerrero-Plata

**Affiliations:** Department of Pathobiological Sciences, School of Veterinary Medicine, Louisiana State University, Baton Rouge, LA 70803, USA; pbabaw1@lsu.edu

**Keywords:** respiratory via infections, coinfection, virus, viral, infectious disease

## Abstract

Respiratory viral coinfections are a global public health threat that poses an economic burden on individuals, families, and healthcare infrastructure. Viruses may coinfect and interact synergistically or antagonistically, or their coinfection may not affect their replication rate. These interactions are specific to different virus combinations, which underlines the importance of understanding the mechanisms behind these differential viral interactions and the need for novel diagnostic methods to accurately identify multiple viruses causing a disease in a patient to avoid misdiagnosis. This review examines epidemiological patterns, pathology manifestations, and the immune response modulation of different respiratory viral combinations that occur during coinfections using different experimental models to better understand the dynamics respiratory viral coinfection takes in driving disease outcomes and severity, which is crucial to guide the development of prevention and treatment strategies.

## 1. Introduction

**Figure 1:**
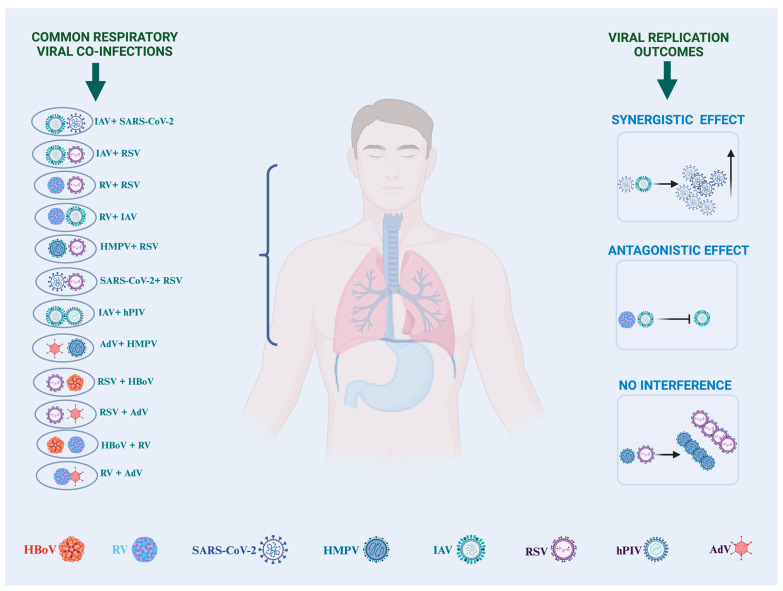
Common combinations of respiratory viral coinfections and viral replication outcomes. RSV: respiratory syncytial virus, HMPV: human metapneumovirus, IAV: influenza A virus, RV: rhinovirus, SARS-CoV-2: Severe Acute Respiratory Syndrome Coronavirus 2, hPIV: human parainfluenza virus, AdV: adenovirus, HBoV: human bocavirus. Created with Biorender.com.

Respiratory viral infections remain a global public health concern as they cause high morbidity and high mortality rates, most especially in vulnerable populations like children, the elderly, and immunocompromised individuals, which may cause economic burdens on individuals, families, and the healthcare infrastructure [1]. One of the challenges of understanding respiratory viral infections is when multiple viruses infect the same host, leading to respiratory viral coinfections, which occur when two or more respiratory viruses are simultaneously present in an individual. Multiple viral infections in the respiratory tract can present overlapping clinical manifestations and lead to different viral replication outcomes depending on whether they interact synergistically, increasing viral replication [2], or can be antagonistic, leading to one virus inhibiting the replication of the other virus [3], or not affecting the replication pattern (Figure 1). 

About 10–20% of all respiratory viral infections involve multiple coinfections [4,5]. A few studies have shown that some coinfections cause alterations in the disease outcome [6], while others indicate that coinfections do not alter the course of the disease [7,8]. These conflicting outcomes obscure the clinical impact of respiratory viral coinfection. Therefore, addressing this ambiguity is crucial for mitigating the heightened disease severity and prolonged illness associated with respiratory tract infections, giving a broader understanding of how respiratory viruses coinfect and unravel the complexities of respiratory viral coinfections. Thus, understanding the intricacies of coinfection can help refine diagnostic methods and integrate advanced molecular techniques to correctly differentiate coinfections from single viral infections.

This review focuses on coinfection by respiratory viruses. It highlights the significance of respiratory viral coinfections based on epidemiological patterns, pathological manifestations, immune response modulation, and the utilization of experimental models that offer a more comprehensive and nuanced perspective on the multifaceted interplay among respiratory viruses, which enhances our understanding of the impact of concurrent viral infections on disease severity and treatment outcomes. 

## 2. Epidemiology

The importance of understanding the dynamics of respiratory viral coinfections lies in their potential and ability to worsen disease outcomes [9], and research has made it clear that respiratory coinfection is now more common than it was thought. The prevalence of coinfection reported in different studies varies depending on several factors, which include geographical location, seasonality, patients’ demographics, and the specific viruses circulating within a given population. However, approximately 1–2 out of every 10 patients diagnosed with respiratory viral infection exhibit coinfection with other respiratory viruses. The use of more sensitive diagnostic methods, such as polymerase chain reaction (PCR) assays [10] and multiplex molecular assays [11], has helped to identify and detect multiple viruses in patients with symptoms of respiratory infection. Although respiratory viral coinfections are detected in smaller proportions of about 20% or less compared to single infections [12,13,14,15,16,17], having a good knowledge of the epidemiology of viral coinfection in different respiratory viruses can be of public health importance as their prevalence may vary across geographical regions. Thus, understanding and monitoring the prevalence of different viral coinfection combinations may help provide the proper intervention through surveillance and targeted prevention and control, as respiratory viral coinfections can affect the effectiveness of vaccination strategies and infection control measures.

The recent coronavirus disease 2019 (COVID-19) pandemic has shed more insight into respiratory viral coinfections as many studies have reported severe-acute-respiratory-syndrome-related coronavirus-2 (SARS-CoV-2) coinfection with many other respiratory viruses [18,19,20,21,22,23], as these coinfections have been attributed to poor disease outcomes [24] and a potential risk factor for disease severity in pediatric patients infected with SARS-CoV-2 [25]. Multiple viruses in an individual can lead to increased hospitalization [26] and disease transmission due to exacerbated viral shedding [27], which puts other uninfected populations at risk of contracting the infection. To better understand how these viruses coinfect, it is fundamental to know the common respiratory viruses and to study the seasons at which they circulate. The most common respiratory viruses are influenza virus type A (IAV) and type B (IBV), parainfluenza virus (PIV) types 1, 2, and 3, respiratory syncytial virus (RSV) type A and type B, the human metapneumovirus (HMPV), rhinovirus (RV), adenovirus (AdV), human coronavirus (HCoV), human bocavirus (HBoV), and enterovirus (EV) [28]. Certain combinations of respiratory viruses, such as IAV, SARS-CoV-2, RSV, RV, HMPV, PIV, HBoV, and AdV, are usually seen in coinfections (Figure 1). In a recent study, an analysis of the prevalence of mono-infections and coinfections of 13 respiratory viruses over 5 years (2013–2018) indicated that respiratory viral coinfections are more predominant in children, especially those under five years of age [29]. 

**Figure 2:**
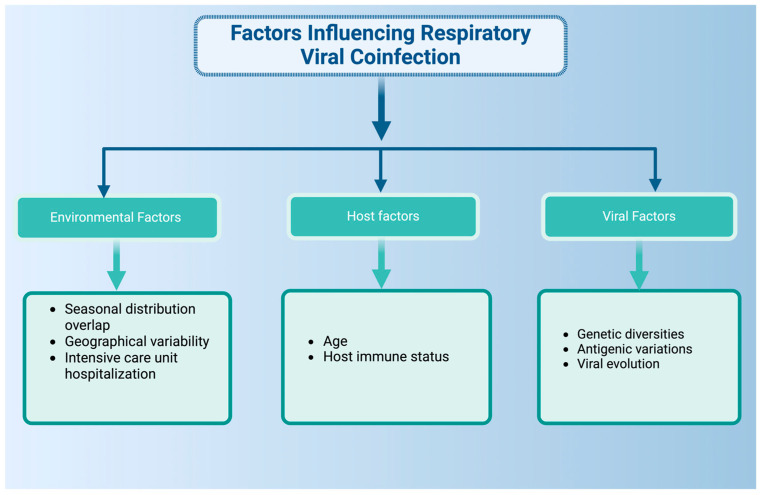
Multiple factors influencing respiratory viral coinfections. Environmental factors, host factors, and viral factors can modulate the coinfection outcome in the host respiratory tract. Created with Biorender.com.

This effect has also been observed in patients infected with SARS-CoV-2, where children infected with SARS-CoV-2 had more respiratory viral coinfections compared to SARS-CoV-2-infected adults, where picornaviruses, RSV, HMPV, AdV, and PIV3 were the most frequently identified coinfecting viruses [21]. The meta-analysis of SARS-CoV-2-positive patients identified IAV, IBV, and RSV as the most commonly identified viruses coinfecting with SARS-CoV-2 [24]. However, the prevalence of respiratory viral coinfections is multifactorial, and additional variables, e.g., seasonal distribution, age, or geographic variability, can influence the coinfection outcome (Figure 2). Studies comparing SARS-CoV-2-positive and negative patients have identified that respiratory viral coinfections with RV, HMPV, PIV, IAV, AdV, and RSV are significantly lower in SARS-CoV-2-positive patients compared to SARS-CoV-2-negative ones [30,31].

Respiratory viral coinfection prevalence is multifactorial. Data analysis about the seasonal variation in virus circulation in Europe, mostly from German university hospitals, indicated that influenza viruses exhibit the highest degree of seasonality infection (~50%), followed by RSV and RV, detected as the most frequent respiratory viral infections. The combination of RV/HBoV exhibited the highest coinfection rate [32].

Bronchiolitis is one of the symptoms of most respiratory tract infections; a study conducted on children with bronchiolitis accessed different infecting viruses and showed that of all the coinfecting pathogens, RSV is the most commonly detected virus, and about one-quarter of children with RSV were coinfected with another virus [10]. The prevalence of RSV in coinfections has been noted in additional studies. Huguenin et al. reported that RSV A and B circulate simultaneously with other respiratory viruses like AdV and PIV3 between November 2007 and February 2008 [33]. Moreover, some reports indicate that RSV frequently coinfects with AdV [33], HBoV [34], and IAV [35,36]. In addition, data from a study from the UK analyzing 4821 specimen results (between 2009 and 2010) indicated that RSV was the most detected respiratory viral infection, while in 13.2% of the samples, a viral coinfection was detected. RV and RSV showed the highest frequency of coinfection with other respiratory viruses, including RSV/RV, PIV/RV, AdV/RV, and PIV/RSV [15].

The interaction among respiratory viruses can be complex and diverse; they can infect and act synergistically [37], which could lead to increased viral replication and the exacerbation of respiratory pathology [38]. They can also act in an antagonistic manner where one virus inhibits the replication of the other or modulates particular aspects of the immune response [39,40]. However, understanding the mechanisms behind these diversities of the synergy and antagonism of viral coinfection remains largely understudied. Using the seasonality of each virus could help predict the pattern of different coinfection pairs and their risk factors, and targeting the driver of the severity of the disease could be a breakthrough for a potential intervention. Knowing the co-occurrences of respiratory viruses could inform us of the common patterns and possibilities of virus pairs in coinfection and help us explore more reasons behind this. However, among clinical studies reporting different respiratory viral coinfections, few have their reports covering the entire respiratory season, and many of the reports have been conducted during different seasons, which may be the reason behind the conflicts in the outcomes observed among viruses that coinfect [41]. Thus, more clinical research capturing entire respiratory seasons is needed to better understand the seasonal variations among coinfections. An improved surveillance system is required to capture more of these trends and help to prevent and manage respiratory viral coinfection cases. 

Genetic diversity and antigenic variations among the strains of these viruses could also contribute to how these viruses coinfect other respiratory viruses [42]. The exchange of genetic materials between two or more viruses is possible during respiratory viral coinfection. Recombination events may impact viral evolution and pathogenesis and lead to novel viral variants with either an enhanced or reduced ability to cause disease. Recombination can also lead to the mutation and shuffling of genetic material between viral strains, generating new combinations of genes and increasing viral genetic diversity. For instance, SARS-CoV itself is a recombination product [43]. In an in vitro coinfection of IAV and RSV, hybrid viral particles and pseudo-type viruses were observed, and they differ structurally from either of the parental viruses [44]. However, extensive research has been documented on the possible changes in the recombination of SARS-CoV-2 with other viruses in humans and other species (Reviewed in [45,46]). Thus, there is a risk of recombination during respiratory viral coinfection, and laboratories need to develop diagnostic tools to detect recombining viruses in patient samples in high-risk areas. Understanding respiratory viral interactions can improve our knowledge of how respiratory viruses recombine and how they can be exploited for epidemiological and medical interventions. As a result, genomic surveillance could be another epidemiological tool that could be used to capture trends of coinfection by using advanced sequencing techniques to characterize co-circulating strains of different viruses involved in coinfection [47]. This can help to track both the patterns and seasons of transmission and shed light on the potential disease outcomes.

## 3. Pathology, Disease Outcomes, and Severity

The impact of respiratory viral coinfection on disease severity and outcome remains largely unclear, and some studies are contradictory to the effects that viral coinfections can cause. Some reports indicate no difference in clinical disease severity between viral coinfections and single respiratory infections, while others report increased severity in coinfection. For example, some reports showed no difference in the clinical symptoms between HMPV and RSV coinfection vs. RSV mono-infection [48,49,50]. In contrast, additional studies indicate an association between HMPV and RSV coinfection and more severe disease or longer symptomatic effects [51,52,53,54]. Conflicting reports like these make conducting further studies into coinfection pathogenesis necessary to better understand the disease dynamics during mono-infection and coinfection. 

Some coinfections may be synergistic and increase the viral load. For instance, in vitro studies using cell lines reported that IAV growth was enhanced by coinfection with human PIV2 [2]. Furthermore, IAV pre-infection strongly enhances SARS-CoV-2 infection by boosting the viral entry and viral load of SARS-CoV-2 in A549 cells. That effect seems specific for IAV since other respiratory viruses like RSV, PIV, and RV do not stimulate SARS-CoV-2 infection. The increased SARS-CoV-2 infection may be influenced by the increased expression of the receptor angiotensin converting enzyme-2 (ACE2) by IAV pre-infection, which allows SARS-CoV-2 virus entry [55]. However, data in vivo indicate that the sequential coinfection of SARS-CoV-2 before IAV in hamsters induced the highest lung viral load compared to IAV before SARS-CoV-2 and mono-infections [38]. 

On the other hand, coinfections may also be antagonistic, causing viral interference during coinfections. This is exemplified by the prior infection of RSV and IAV (H1N1), which inhibited RV replication in a three-dimensional airway epithelial cell model [38]. A similar effect was also observed when RSV pre-infection interfered with HMPV replication [56]. During respiratory viral coinfection, the total viral load during a single infection may differ from that during multiple viruses. That is the case for AdV and PIV1 infections, where viral loads for these viruses decreased in children with multiple respiratory viral infections [17]. Studies using primary human airway cells also showed that prior infection with RV led to a reduced IAV infection and an increased expression of IFN-stimulated genes (ISGs), which could induce the antiviral state [57]. Thus, suppressing one viral infection with another could decrease the prevalence of more pathogenic respiratory viruses at the population level. However, the effect on viral titers during a respiratory viral coinfection is deemed to be more complex as data reported in the Syrian hamster model indicate that animals pre-infected with IAV before SARS-CoV-2 showed lower SARS-CoV-2 viral loads but increased IAV titers [38,58]. In contrast, the coinfection of SARS-CoV-2 and IAV in mice led to the prolonged persistence of these viruses in the lungs [59].

Respiratory viral coinfections also impact the disease pathogenesis and hospitalization rates compared to single respiratory viral infection [60], suggesting an increased disease severity and outcomes during coinfections. It has been found that there is a significantly higher risk of death in children with respiratory viral coinfections compared to children with single infections [8], which makes it very important to understand the factors that contribute to these severities. However, the mechanisms and factors influencing the disease outcome and severity are largely unknown. A clinical study that compared SARS-CoV-2-infected patients with or without viral coinfections reported that the clinical features and prognosis of the two groups are similar [61]. However, data meta-analysis on the clinical severity of COVID-19 patients with IAV and SARS-CoV-2 coinfection indicates that this coinfection might be associated with a higher risk of intensive care unit (ICU) admissions and mechanical ventilation but does not increase the risk of death [62]. Another clinical report showed that SARS-CoV-2-infected patients aged 65 and above have a worse clinical outcome when coinfected with other respiratory viruses [63]. Overall, SARS-CoV-2-infected individuals coinfected with other respiratory viruses suffer from dyspnea compared to SARS-CoV-2 mono-infected patients [9]. The negative effect of this has been reproduced in the mouse model, where pre-infection with IAV before the subsequent infection of SARS-CoV-2 caused more severe lung pathology [55]. A similar effect was observed in hamsters coinfected with IAV and SARS-CoV-2, where the coinfection led to more severe disease [38] and prolonged pneumonia [64]. Coinfection studies of RSV and IAV showed changes in weight loss and airway obstruction. Increased survival was observed with RSV pre-infection before an IAV infection compared to IAV infection alone. The protection was observed even when the interval from RSV pre-infection to IAV infection was up to 30 days apart [65].

In contrast, pre-infection with IAV, before RSV, led to increased morbidity and mortality similar to IAV mono-infection [66]. This further shows that the order in which respiratory viruses coinfect plays an important role in disease outcome and severity. Another study shows that in IAV and RSV coinfection, IAV replication has a higher titer while RSV replication is reduced [44]. This increase in IAV replication kinetics observed in RSV coinfection contrasts with what is observed when IAV coinfects with RV, where there is an inhibition of IAV replication when in association with RV [57]. Likewise, mice infected with RV were protected from the lethal infection of Beta CoV, with observed reduced disease and pulmonary inflammation [67]. This indicates that the outcomes of coinfections are highly dependent on virus combinations as they trigger virus-specific responses. The coinfection of IAV and hPIV2 has also been reported, and it was found that hPIV2 infection enhanced the growth of IAV, likely because hPIV2 can induce cell-to-cell fusion through its F-protein. However, IAV infection did not affect the growth of hPIV2 [2].

RSV and HMPV coinfection in infants have been associated with severe bronchiolitis and an increased risk of admission into the pediatric intensive care unit [51]. This effect was also found in a systematic review where the highest clinical severity was found when RSV and HMPV were coinfected [53]. However, data using the ex vivo 3D air–liquid interface epithelial cell culture indicated that the pre-infection of RSV blocked the replication of HMPV. In contrast, RSV replication was not affected by initial infection with HMPV [56]. In a mathematical model that simulates the coinfections of some respiratory viruses like SARS-CoV-2, IAV, RSV, and RV, it was found that RSV had the most impact on the prevalence of SARS-CoV-2 when the two viruses co-circulated and that the coinfection of RSV with SARS-CoV-2 caused the largest suppression of SARS-CoV-2 infection [68]. This effect may be caused by the pre-infection of RSV, leading to the decreased replication of SARS-CoV-2 and clinical disease [69], which could mean that RSV provides some protection to SARS-CoV-2. This may be why, although there is an increase in hospital stays in patients with RSV and SARS-CoV-2 coinfection, there is no need for intensive care or mechanical ventilation [70]. Additional respiratory viral combinations have been reported to worsen the disease outcome (Table 1). Overall, the clinical effect of respiratory viral coinfections is obscured by divergent differences in pathology between the different virus combinations observed, and further research is needed to determine the mechanisms underlying the detrimental effects of respiratory viral coinfections. 

## 4. Modulation of Immune Response to Different Combinations of Respiratory Viral Infection

The immune response plays a crucial role in combating respiratory viral infection; however, during coinfection, the host immune system may present a different response and add more complexity to the immune cell activation and cytokine production [73]. As a result of the complex interaction between coinfecting viruses, which may be synergistic or antagonistic, the coinfection of these viruses may alter and modulate the induction pattern of cytokines and chemokines [74,75]. An in vivo study of IAV and SARS-CoV-2 coinfection in hamsters showed a significant increase in serum interleukin-6 (IL-6), which is a cytokine that plays a crucial role in the progression of inflammation and could be involved in the observed increased severity of pneumonia [64]. In another in vivo study with mice, IAV and SARS-CoV-2 were coinfected sequentially; the single infection of IAV caused increased levels of tumor necrosis factor-alpha (TNF-α), interleukin-1 alpha (IL-1α), IL-6, and interferon beta (IFN-β), while single infection with SARS-CoV-2 caused moderate increases in IL-1α, IL-6, and IFN-β, lower than those observed in IAV single infection. However, when they are coinfected, and IAV infects first before SARS-CoV-2, there is a little increase in the inflammatory cytokines, but when SARS-CoV-2 comes first before IAV, it leads to a rapid increase in TNF-α, IL-1α, IL-6, and IFN-β, levels [59]. This suggests that the virus infecting first during coinfection influences the regulation of immune response.

For RSV and IAV coinfection studies in mice, TNF-α and IL-6 were induced during IAV mono-infection. However, a very low amount of these cytokines was observed during RSV mono-infection. When the mice were infected first with RSV and then 30 days later with IAV, they showed a significantly reduced level of TNF at day 4 and IL-6 at days 2 and 4 after the IAV infection [65]. This study suggests that RSV induces protection against subsequent IAV coinfection and that the innate immune response plays a role in mediating immune response during viral coinfection. In a study that reported that prior RSV infection blocked the replication of HMPV, they showed that the interaction between HMPV and RSV was disrupted in the absence of a primary epithelial interferon (IFN) response, which resulted in the reduced inhibition of HMPV replication and increased HMPV infection in the coinfected cells, suggesting IFN’s involvement in RSV blockage of HMPV replication [56]. Another report showed that, based on the clinical history and cytokine profile between RSV and HMPV, it is difficult to distinguish infants with severe bronchiolitis caused by RSV mono-infection or coinfection with HMPV [52]. This is because similar cytokines were produced during a mono-infection of both viruses, even though RSV induces a higher amount of those cytokines. These differences in the immune response show that the immune response during respiratory viral coinfection is a complex and dynamic process. Further research on respiratory viral coinfection is needed to better understand the immune response and the host–pathogen interaction. Therefore, elucidating the interplay between the coinfecting viruses can help to better guide our understanding and proffer effective diagnosis and prevention strategies against coinfection.

## 5. Experimental Model for Studying Respiratory Viral Coinfections

Experimental models play a crucial role in advancing our understanding of respiratory viral coinfection as they provide valuable insights into the pathogenesis of coinfections and immune modulation stimulated by different virus combinations to better understand the pathology and the effect of coinfection on the modulation of disease outcomes during respiratory viral coinfection [76].

Animal models, such as mice, hamsters, and ferrets, are the most explored model systems for the in vivo study of respiratory viral coinfections. The mouse model has been extensively used to study the coinfection of multiple respiratory viruses, including IAV and RSV [66,77]. The humanized mouse model has been a valuable tool for studying SARS-CoV-2 coinfections since mice are not naturally susceptible to SARS-CoV-2 because they do not express the human angiotensin-converting enzyme 2 (ACE2) receptor necessary for SARS-CoV-2 cell entry [78]. Therefore, transgenic mouse models expressing the human ACE2 receptor driven by cytokeratin-18 (K18-hACE2) allowed the study of SARS-CoV-2 infection to recapitulate several aspects of lung inflammation observed in severe and non-severe COVID-19 manifestations [79,80]. The viral interference between IAV and RSV has also been studied in ferrets [39] since this model recapitulates the symptoms of IAV in humans [81]. Hamsters are another model that has been used for several models of respiratory viral coinfections [38,58,64]. Likewise, rats have also been modeled to study subtypes A and B of RSV coinfection [82]. However, one of the challenges of animal studies is applying the model correctly because a human virus may interact differently when used to infect animals [83]. In addition, the results of mono-infections versus coinfections, such as the pathology and disease outcomes in animal models, may not be translated to what is observed in natural infection in humans [84]. Therefore, it is important to use or develop an experimental model that more closely recapitulates and mimics the complexity of the human respiratory tract to resemble the pathophysiology of the host–pathogen interaction.

Coinfections have also been extensively studied in vitro using immortalized cell lines [85,86]. However, cell monolayers do not capture the processes that occur at the tissue level, and they may be insufficient for predicting the impact of respiratory viral coinfections in humans [87,88]. Therefore, more physiological primary cell culture models have been developed to create a more appropriate experimental model. Primary human epithelial cells grown in an air–liquid interface (ALI) to generate a pseudostratified epithelium represent a more physiological model that closely mimics the human airway epithelium [89]. This model has been explored in coinfection models to study the interaction of IAV + RSV [44,90], HMPV + RSV [56], IAV + SARS-CoV-2 [91], and SARS-CoV-2 with several other respiratory viral infections (RV, RSV, HMPV, IAV) [92].

Nevertheless, this model is faced with some disadvantages, one of which is the fact that this culture is limited to epithelial cells and does not allow the exploration of the contribution of other cells in the respiratory system. Therefore, the use of lung organoids, e.g., derived from human pluripotent stem cells, provides a model with multiple cell types, which offers a system that recapitulates key aspects of the lung physiology critical to studying the mechanisms and effects of respiratory viral coinfections and potential therapeutic molecules [93]. Although lung organoids are not without experimental limitations, they have been used to study the molecular mechanisms underlying enhanced disease with the coinfection of IAV and SARS-CoV-2 [94]. Future research, however, is needed to improve this in vitro system and overcome the technical limitations of viral inoculation in lung organoids.

Besides physiological models, mathematical models have also been used to contribute to understanding coinfection dynamics. These models capture the transmission dynamics during coinfections [95] by incorporating viral transmissibility, individual susceptibility, and interactions between coinfecting strains and also integrating epidemiological, virological, and immunological factors to analyze the disease outcomes of multiple virus combinations during viral coinfections. A mathematical model has been used to simulate co-circulating respiratory viruses, such as SARS-CoV-2, IAV, RSV, and RV, where it was found that the coinfection of RSV with SARS-CoV-2 can cause the highest suppression of SARS-CoV-2 [68]. Likewise, the dynamics of coinfection of IAV and RSV, RV, PIV, and HMPV have been explored through a mathematical model, where the viruses fall for resource competition [96]. However, mathematical models also have limitations, like predicting the immune response inaccurately [97]. Nevertheless, mathematical modeling also offers a powerful tool for investigating the complexities of respiratory viral coinfection through available information and the basic knowledge of the biological mechanism these viruses use to cause infection and how this infection spreads in the population [98].

## 6. Future Perspectives

The interaction between respiratory viruses occurs at multiple levels, from populations to individuals and tissues, with quantifiable outcomes. However, more research is needed to better understand how multiple viruses coinfect and drive disease outcomes, which can improve diagnostic and therapeutic approaches and targeted prevention strategies. Some of the more sophisticated methods for the confirmatory diagnoses of respiratory viral coinfections are hybrid-capture sequencing platforms like Virome Capture Sequencing for Vertebrate Viruses (VirCapSeq-VERT), which is a technique that is particularly useful in the study of complex viromes, where multiple viral species may co-exist and has been used to determine the rate of respiratory viral coinfections with SARS-CoV-2 [99], and the Twist Respiratory Virus Panel, which can screen 29 human respiratory viruses including coronaviruses, enteroviruses, paramyxoviruses, pneumoviruses, influenza viruses, and adenoviruses [100], as powerful tools that can be explored in determining respiratory viral coinfections.

Understanding how viral interactions occur in coinfections can help researchers unravel specific signatures, distinguishing viral combinations from single infections. Knowledge of the replication kinetics and immune response during coinfections can provide insights into the infection pattern of respiratory viruses when they coinfect a susceptible host. Several models, such as the human ALI culture and lung organoids [101], have been developed to improve the study of viral coinfections. They represent a more physiological respiratory system to study respiratory infections in humans. However, limitations remain for the current experimental models, such as a lengthy process to generate the systems, genetic variability, cell maturity, or organoid survival. Therefore, developing novel physiological models, such as organotypic human lung buds [102], or improving the current models is warranted.

Furthermore, studying the long-term effects of coinfections is crucial to designing novel targeted antivirals to provide broader protection against multiple respiratory viruses, which can prevent excessive inflammation and help reduce tissue damage. Overall, the knowledge generated in human respiratory physiological systems can help develop better strategies to combat the effects of coinfecting viruses in the respiratory tract.

## Figures and Tables

**Table 1:** Overview of viral coinfections and their associated disease outcomes.

Respiratory Viral Coinfection	Disease Outcome	References
HMPV and RSV	Severe bronchiolitis,	[51]
	Increased chance of admission into the pediatric intensive care unit	
	Pneumonia	[71]
IAV and SARS-CoV-2	Severe and prolonged pneumonia	[64]
	Alveolar necrosis	[55]
RSV and IAV	Severe airway obstruction	[65]
HMPV and AdV	Bronchitis, Bronchiolitis, Asthmatic bronchiolitis	[71]
RSV and RV	Fever and hypoxia,	[72]
	Pediatric intensive care unit admission	
